# My brand is +: when intersecting regularities transfer polarities instead of valence

**DOI:** 10.1007/s00426-026-02429-9

**Published:** 2026-11-12

**Authors:** Marcel Pauly, Lea Marie Siegel, Dirk Wentura

**Affiliations:** Department of Psychology, Saarland University, Campus A2 4, 66123 Saarbrücken, Germany

## Abstract

Recent research suggests that valence can be transferred from one category to another through intersecting regularities (IR). For example, if two categories share a common response key, the valence of a source category (e.g., positive stimuli) can be transferred to a target category (e.g., a previously neutral brand). The present study investigates whether polarity differences of the source categories (i.e., asymmetric processing of these categories; Proctor & Cho, 2006) could be the underlying mechanism for differences in indirect and explicit measures. This hypothesis is motivated by the fact that the Implicit Association Test (IAT) is most often used as an indirect measure in IR tasks and is known to be susceptible to asymmetric processing. In other words, apparent valence differences between target categories could actually be artifacts arising from asymmetric processing of those categories, induced by asymmetry in the source categories. For explicit measures of valence, any observed differences would reflect genuine effects but might still be driven by the asymmetric processing of the source categories. Across four experiments, the polarities of the source categories were manipulated. The results show that polarity framing influenced IAT performance in Experiments 1 and 2. An alternative indirect measure—the Affect Misattribution Procedure—did not reveal valence differences (Experiment 3). The critical tests of the manipulation on explicit ratings were not significant in any individual experiment. However, a cross-experiment analysis revealed cautious evidence for a small effect.

## Public significance statement

Researchers often assess attitudes through direct ratings and indirect tasks, such as the Implicit Association Test. Across four experiments, we examined whether changing how categories are framed during a learning task influences later responses, finding effects on Implicit Association Test performance and cautious evidence for a small effect on direct ratings. These findings suggest that interpreting attitude measures requires considering both changes in evaluation and structural differences in how people process the categories involved.

It is a long-standing topic in psychology whether likes and dislikes toward persons, groups, objects, or brands can result from relatively unobtrusive learning procedures—that is, procedures occurring outside of explicit communicative acts (e.g., “This is Jim. Jim has murdered his wife. How do you like Jim?”). Two well-known examples are evaluative conditioning (e.g., De Houwer et al., 2001) and mere exposure (Bornstein & Craver-Lemley, 2022; Zajonc, 1968). In evaluative conditioning, initially neutral stimuli are paired with (or presented in close succession to) positive or negative stimuli, which—under specific conditions investigated in related research—induces assimilative valence changes in the neutral stimulus.

In recent years, another procedure—known as *intersecting regularities*—has been proposed. Several variants of this procedure exist. For example, Hughes et al. (2016) instructed participants to respond to stimulus S1 by pressing one key (R1) and to stimulus S2 by pressing a different key (R2). In some trials, following a response to S1, a positively connoted picture was presented (O1); in other cases, a new, initially neutral brand logo was shown (O2). When participants responded to S2 with R2, either a negative picture (O3) or another neutral brand logo (O4) was displayed. Attitudes toward the two brands were then measured. The Implicit Association Test (IAT; Greenwald et al., 1998) was typically used as an indirect measure of attitude, while a behavioral choice task and explicit ratings served as direct measures. The results showed that the brand associated with the positive outcome O1 via R1 received significantly higher liking than the brand associated with the negative outcome O3 via R2.

In another version of the task, Ebert et al. (2009) used the brands *Haribo* and *Milka* (well-known German confectionery brands) as target categories. Participants were instructed to respond to images of *Haribo* or *Milka* products using two keys, and to categorize positive or negative words with those same keys. In one condition, *Haribo* and positive words (and *Milka* and negative words) shared the same response key. In the other condition, this assignment was reversed. The study found that the product sharing a response key with positive words was subsequently evaluated more positively than the product paired with negative words. The authors assessed liking by using an evaluative priming task (Draine & Greenwald, 1998) and a Go/No-Go Association Task (Nosek & Banaji, 2001), a variant of the IAT.

Hughes et al. (2016) refer to this form of evaluative learning as intersecting regularities (IR). According to this account, participants perceive the relationship between R1 and O1 as a regularity because pressing R1 occasionally results in the presentation of the positive outcome O1. In addition, the relationship between R1 and O2 constitutes a further regularity: if R1 follows S1, it can also lead to the presentation of the neutral brand O2. Thus, two regularities intersect in relation to R1. Because both outcomes are linked to a common feature (R1), it is assumed that the positive valence of O1 (the source category) is transferred to the initially neutral brand O2 (the target category). The same reasoning applies to the relationships R2 → O3 and R2 → O4, whereby the negative valence of O3 is transferred to O4. The principle of intersecting regularities can also be applied to the experiment by Ebert et al. (2009). One regularity here is “positive word, press key A,” and the other is “*Haribo* brand, press key A.” Here too, positive words and *Haribo* share the same response, which is assumed to increase the positive evaluation of *Haribo*. Conversely, *Milka* is evaluated more negatively because it shares a response feature with negative words.

## Self-referencing task

A variant of the IR task by Ebert et al. (2009) is the self-referencing (SR) task. Richetin et al. (2016) introduced this paradigm with two fictitious brands, *Ambio* and *Ecove*. In a preliminary learning phase, participants were instructed to respond to various products from these brands to become familiar with the brand logos. In the subsequent SR task, participants responded to four categories: two target categories (the brands Ambio and Ecove) and two source categories—self-related words (e.g., “self,” “me,” “my”) and other-related words (e.g., “they,” “their,” “them”). In this paradigm, one target and one source category shared a common response. For example, participants were instructed to press R1 when presented with an Ambio product or a self-related word, and to press R2 when presented with an Ecove product or an other-related word. The assignment of target to source categories was counterbalanced across participants. After the SR task, brand preferences were assessed using both an indirect measure and explicit ratings. First, an IAT was administered in which positive and negative words served as attribute categories, and Ecove and Ambio served as target categories.^1^

Participants then completed a hypothetical shopping task, choosing between four Ambio and four Ecove products. Finally, they rated the products on four dimensions (unattractive–attractive, unpleasant–pleasant, worthless–valuable, useless–useful) using a seven-point scale. Richetin et al. (2016) found that, in both explicit ratings and the indirect measure (i.e., the IAT), participants preferred the brand previously associated with the self. This preference was also evident in the hypothetical shopping task.

The results of this and related experiments (Mattavelli et al., 2019, 2021; Perkins & Forehand, 2012; Prestwich et al., 2010) are typically explained by the presumed positivity of the self. In general, the self is regarded as a positive source category; consequently, a neutral stimulus (such as the brand Ambio in Richetin et al., 2016) is expected to be evaluated more positively than a comparison stimulus due to its shared response feature with the self. A substantial body of literature has examined how the self influences and shapes attitudes toward other entities. The prevailing view is that healthy adults exhibit a positivity bias—a tendency to evaluate their own traits, behaviors, and thoughts more favorably than those of others (Mattavelli et al., 2017; Yamaguchi et al., 2007).

## The IAT as a measure of indirect liking

In the aforementioned studies, implicit evaluations are frequently measured using the IAT. Mattavelli et al. (2017) published an overview summarizing results from research based on the self-referencing task. In this article, they reviewed experiments from their own lab, analyzing data from 53 studies, in 50 of which the IAT was employed as an indirect measure. Notably, other studies employing the SR task (Perkins & Forehand, 2012; Prestwich et al., 2010) likewise used the IAT to assess implicit preferences. More generally, the IAT is a widely used tool for assessing implicit attitudes (Bar-Anan & Nosek, 2014).

The standard IAT consists of several blocks in which participants categorize stimuli belonging to four concepts—two target categories and two attribute categories—that are mapped onto two response dimensions. In the present context, for example, the neutral brands Ambio and Ecove could serve as the target categories, while positive and negative words serve as the attribute categories. In the critical blocks, one target category and one attribute category are assigned to the same response key, while the remaining two categories are assigned to the alternative key (e.g., Ambio and positive words → left key; Ecove and negative words → right key). In a subsequent block, the assignment is reversed (e.g., Ambio and negative words → left key; Ecove and positive words → right key). If Ambio has acquired a positive valence during the preceding IR task, responses should be systematically faster in the block where Ambio and positive attributes share a response key than in the block with the reversed assignment.

## A working hypothesis: polarity induction instead of valence induction

The predominance of the IAT as the indirect measure in the SR task suggests an alternative interpretation of the processes underlying the reported effects. Briefly stated, the standard interpretation of the SR effect assumes that the SR task shifts the valence of the a priori neutral brand names, which is subsequently detected by the IAT. In contrast, the alternative interpretation posits that during the SR task, the initially symmetrical comparison of the brand names becomes asymmetrical —or “polar,” according to the Polarity Correspondence Principle (PCP; Proctor & Cho, 2006). According to this view, the IAT does not capture task-induced valence differences, but rather reflects structural polarity differences between the categories (Rothermund & Wentura, 2001, 2004; Proctor & Cho, 2006).

This working hypothesis comprises three components. First, the self/other dichotomy (i.e., the source dichotomy in the SR task) is plausibly an asymmetric, “polar” dichotomy in the sense of Proctor and Cho (2006), meaning it is asymmetrically coded. One pole of this contrast (self) corresponds to the + polarity, while the other pole (other) corresponds to the – polarity. Importantly, + and – in this context should not be confused with positive and negative affective valence. Polarity can be understood in terms of binary contrasts where one pole is defined by the presence of a feature and the other by its absence. For example, if participants categorize objects as “can fly” versus “cannot fly,” objects that can fly—those possessing the defining feature—constitute the + polarity category, whereas objects lacking the feature constitute the – polarity category. By analogy, the binary distinction between self and other can be considered polar asymmetric: categorizations are more of the type “yes, self” (+ polarity) versus “no, not self” (– polarity). Pauly and Wentura (2025) provided evidence for this assumption.

Second, when an a priori neutral, unfamiliar, and thus symmetric dichotomy (e.g., Ambio vs. Ecove) is mapped onto the same response keys as a polar dichotomy (i.e., self/other), this target contrast itself becomes asymmetric. The brand sharing a response key with the + polarity source category (i.e., self) acquires + polarity, whereas the brand sharing a response key with the − polarity source category (i.e., other) acquires − polarity. Consequently, by the end of the IR task, the products are processed more in terms of an asymmetric contrast (e.g., Ambio vs. non-Ambio) than a symmetric comparison (Ambio vs. Ecove).

Third, it is well established that the IAT is sensitive to polarity correspondences (Rothermund & Wentura, 2001, 2004; Proctor & Cho, 2006). If both category dichotomies used in the IAT—the typical positive vs. negative attribute dichotomy and the target dichotomy (here, *Ambio* vs. *Ecove*)—can be considered polar, responses in the block where corresponding polarities (i.e., +/+ and −/−) share a response key will be faster than responses in the block where the corresponding polarities are assigned to different keys.

We illustrate the polarity interpretation of the IAT using an experiment by Mitchell (2004). The author used two object categories in an IAT characterized as “[object] can fly” (e.g., kite) and “[object] has teeth” (e.g., shark), respectively. Notably, the two object sets were non-intersecting: objects that could fly had no teeth, and objects with teeth could not fly. All objects were used as target stimuli in an IAT, with positive and negative words serving as attribute categories. Half of the participants categorized the objects as “can fly” versus “cannot fly,” using the same keys as for the positive/negative attribute categorization; the other half categorized the objects as “has teeth” versus “has no teeth.” In both samples, responses were faster in the block in which the target affirmation (i.e., “yes, can fly” or “yes, has teeth”) shared a response key with the positive attributes. The standard interpretation of the IAT in terms of valence connotations seems problematic here, since the same objects that appear positively connoted in one version of the IAT appear negatively connoted in the other. An interpretation in terms of category asymmetries, however, is straightforward: participants categorize the targets according to whether they, for example, can fly (+) or cannot fly (–). The positive/negative comparison can also be understood as evaluating whether the word is + or not (–). This means that the categories “can fly” and “positive” are reference categories (and thus +) against which the other categories are evaluated (which are then –). The IAT effect can therefore also be explained on the basis of the PCP: if the two + coded/– coded categories share a response, participants respond faster than if there is a mismatch between target and attribute polarities. In the former case, participants can simplify the task by mapping one key onto “+” polarity and the other onto “-” polarity, a cognitive simplification that is unavailable in the reversed block.

Rothermund and Wentura (2001, 2004) provided several experiments to corroborate this interpretation. For instance, they conducted two versions of an ageism IAT—a paradigm that, under the standard valence account, assesses implicit prejudice against older adults—using young and old first names as target categories. In one version, positive and negative words served as attribute categories. As expected, the block in which young names and positive words shared a response key (while old names and negative words shared the alternative key) produced faster responses than the block with the reversed assignment. Although this finding is compatible with the hypothesis that the category “old” is negatively connoted and “young” is positively connoted, their second version challenged this purely affective account.

In this second version, neutral words versus non-words served as attribute categories. Crucially, the block in which young names and words shared a key, and old names and non-words shared the alternative key, yielded significantly faster responses than the reversed block. This pattern does not imply that the category “young” is conceptually associated with “wordiness” or “old” with “non-wordiness.” Rothermund and Wentura (2001, 2004) explained this pattern in terms of corresponding figure–ground asymmetries: “old”, “negative”, and “non-word” served as salient figure categories, whereas “young”, “positive”, and “word” served as ground categories. Their explanation therefore concerned corresponding salience asymmetries rather than conceptual associations between age and lexical status.

To avoid confusing readers, we should briefly address the fact that Rothermund and Wentura (2001, 2004) did not use the terminology of polarity at the time. The essence of their approach, however, is that binary decisions are (often) processed asymmetrically. If the asymmetries associated with the two dichotomies in the IAT are aligned in their mapping onto the same response keys, responses are faster than when they are not aligned. This account is fully compatible with the polarity correspondence principle (Proctor & Cho, 2006; Proctor & Xiong, 2015; see also Kinoshita & Peek-O’Leary, 2006, for a further discussion of this issue).

The present study now examines the question of whether in some cases instead of valence, polarity assignments are transferred to a target category. To illustrate this mechanism, we refer back to the study by Richetin et al. (2016), in which the SR task was used to establish presumed valence differences between the two initially neutral brands Ambio and Ecove. Assuming that the self-category is coded with + and the other category with – and that Ambio is paired with the self-category and Ecove with the other category, the SR task could result in the initially non-polar brand Ambio being + coded and Ecove being – coded. When subsequent evaluations are assessed via the IAT—where positive words serve as the + coded reference category and negative words as the – coded category—an IAT effect may emerge that is not driven by genuine valence differences between Ambio and Ecove, but rather by structural polarity differences between the two brands. Consequently, participants respond faster when the two + coded (and – coded) categories share a key (Ambio/positive, Ecove/negative) than when they do not.

## Explicit measures and alternative indirect measures

As a rule, IR studies utilize both indirect measures and explicit ratings to assess changes in preferences (e.g., Mattavelli et al., 2017, 2019, 2021; Perkins & Forehand, 2012; Prestwich et al., 2010). The polarity correspondence principle makes no assumptions about how the encoding of stimuli might influence explicit preferences. Nevertheless, since IR tasks typically employ both indirect measures and explicit ratings, it is important in the context of the current paper to collect explicit measures as well, to examine whether polarities can also have an effect.

It is possible that polarity coding affects not only the IAT but also explicit evaluations. Such a finding would provide initial evidence that polarity coding influences not only cognitive processes involved in response selection—and thus reaction times—but also evaluative judgments.

Our basic assumption is that the IR task may establish one brand as the + pole of the brand dichotomy. Although Proctor and Cho (2006) do not propose that positive-polarity categories are inherently easier to process, assigning a brand the status of the reference (+) category may nevertheless increase its processing fluency, its salience, and its association with affirmative responses. Each of these consequences could, in principle, contribute to more positive evaluations.

With regard to processing fluency, it has long been known that stimuli that are processed more fluently tend to be evaluated more positively (e.g., Reber et al., 1998; Winkielman & Cacioppo, 2001). If the IR task induces an (implicit) task recoding from, for example, “Ambio versus Ecove?” to “Ambio versus not Ambio?” (with Ambio serving as the induced + category), Ambio becomes the reference category against which all stimuli are evaluated. Consequently, Ambio stimuli may become more efficient retrieval cues for the corresponding memory representation than Ecove stimuli, potentially resulting in more fluent processing. This increased processing fluency may, in turn, produce slightly more positive explicit evaluations.

With regard to salience, Mrkva and Van Boven (2020) proposed that increased salience contributes to the well-established mere exposure effect, whereby repeated presentation of a neutral stimulus increases its likability (Zajonc, 1968). According to their account, repeated exposure enhances a stimulus’s salience, making it stand out relative to less frequently presented alternatives. They found that salience ratings statistically mediated the effect of exposure frequency on liking, and in a final experiment, directly manipulating salience again increased liking. Although both brands are presented equally often in the IR task, polarity induction may nevertheless increase the subjective salience of the + category, causing it to stand out relative to the − category and thereby producing a slight evaluative advantage.

Finally, if the IR task induces an (implicit) task recoding, one brand becomes consistently associated with affirmation (e.g., “Yes, this is Ambio”), whereas the other becomes associated with negation (e.g., “No, this is not Ambio”). Such an asymmetry in affirmative versus negative responding may itself contribute to small differences in explicit evaluations. At present, however, this possibility remains speculative, as we are unaware of studies that have directly examined whether repeated associations with affirmative rather than negative responses influence subsequent evaluations.

In some studies, alternative indirect measures have been employed instead of the IAT. In their overview, Mattavelli et al. (2017) also reported the use of the Affect Misattribution Procedure (AMP; Payne et al., 2005) as an indirect measure. The theoretical assumptions formulated for explicit ratings can be applied equally to these alternative indirect measures: Valence differences may be induced via the asymmetric processing of distinct stimulus categories. If polarity framing changes the evaluative responses relevant to another indirect measure, an effect may also emerge on that measure. Conversely, if asymmetric processing does not modulate underlying stimulus valence, then indirect measures other than the IAT should remain unaffected by polarity manipulations.

## The present study

In the current research, we aimed to investigate the assumed influence of polarity encodings induced by the intersecting regularity (IR) task on both indirect and explicit liking. To this end, we conducted four experiments. In Experiment 1, we sought to demonstrate that IAT scores for two brands can appear to reflect valence connotations under standard interpretations, but instead could reflect task-induced polarity asymmetries. We used two word lists as the source dichotomy in the IR task. In close correspondence to Mitchell (2004; see above), the lists consisted of two sets of items that could be categorized from two perspectives: “animals that can swim” versus “animals that can fly,” where all swimming animals were non-flying and vice versa. Half of the participants categorized the items as “can swim” versus “cannot swim,” while the other half categorized them as “can fly” versus “cannot fly.” This manipulation was intended to induce specific polarity codings for the word lists: for one half of the sample, swim words were + polarity and fly words – polarity, and for the other half, swim words were – polarity and fly words + polarity. In the subsequent IR task, swimming animals were paired with one brand (hereafter referred to as the swim brand) and flying animals with the alternative brand (hereafter referred to as the fly brand).

Following the IR task, participants completed an IAT with positive and negative words as attribute categories and the two brands as target categories. We calculated the IAT effect by arbitrarily defining the block in which the swim brand and positive words shared a response key as the “compatible” block, whereas the block in which the swim brand was paired with negative words was defined as the “incompatible” block. Consequently, a positive IAT score reflects an implicit preference for the swim brand over the fly brand, whereas a negative score reflects the opposite pattern.

If polarity coding drives the effect, the IAT outcomes should reverse depending on which source category is coded as + polarity. Specifically, participants for whom swim words are + (swim = +) should show a positive IAT effect, whereas participants for whom swim words are – (fly = +) should show a negative IAT effect. An account based solely on transferring pre-existing valence from the normatively neutral source categories would not predict this difference. However, this does not exclude the possibility that polarity framing itself induces evaluative changes that subsequently contribute to the IAT effect (see *General Discussion* for a more detailed elaboration of this point).

In Experiment 2, we aimed to investigate the same effect using an SR task. Instead of swimming/flying animals, self- or other-referential stimuli served as the source categories. We again manipulated category polarities: one half of the participants were instructed to respond to whether the source stimuli were self-relevant (self = +), while the other half responded to whether the stimuli were other-relevant (other = +). Analogous to Experiment 1, we calculated IAT effects such that positive values indicate a preference for the brand paired with the self and negative values indicate a preference for the brand paired with the other. If polarity coding drives IAT effects, we expected a positive IAT score in the self = + condition and an at least significantly smaller effect in the other = + condition. (For an explanation of why we do not expect a complete reversal of the effect, see Discussion of Experiment 1.)

Moreover, we measured explicit preferences for both brands in Experiments 1 and 2 after administering the IAT. If explicit ratings are influenced by stimulus polarity, we would expect to find an interaction between the + polarity assignment and brand type. Specifically, in the swim = + condition (and the self = + condition in Experiment 2), the swim or self-brand should be explicitly preferred over the fly or other-brand. Conversely, in the fly = + condition, preferences for the brands should reverse, whereas in the other = + condition, preferences for the self-brand should be significantly attenuated.

Experiments 3 and 4 served as conceptual replications of Experiments 1 and 2, respectively, but omitted the IAT as the indirect measure. In Experiment 3, we again utilized swim versus fly words as the source categories, but this time employed the AMP to assess implicit evaluations, followed by explicit ratings. This experiment aimed to test whether (a) an indirect measure that cannot be easily reinterpreted by the PCP is still influenced by polarity manipulations and (b) whether effects on explicit ratings can be observed in the absence of a preceding IAT. Experiment 4 similarly tested the influence of polarity on explicit ratings, this time using the self- versus other-referential dichotomy.

## Experiment 1

In Experiment 1 we tested the effect of polarity coding in an IR task using *Ambio* and *Ecove* as neutral brands. In accordance with the majority of studies, we used an IAT as the indirect measure. We expected the IAT to reflect polarity differences between the target categories. We further used self-reported evaluations as explicit measures, which were used in other studies as well (e.g., Mattavelli et al., 2019; Perkins & Forehand, 2012; Richetin et al., 2016).

### Method

#### Transparency and openness

We report how we determined our sample size, any data exclusions, all manipulations, and all measures in this study and we follow journal article reporting standards (Kazak, 2018). Raw data, analysis scripts and experiment files for each experiment can be found at https://osf.io/ev89m. All experiments were pre-registered before data were collected. Preregistration for Experiment 1 is available under: https://aspredicted.org/q52m-s9mq.pdf. Data were analyzed using IBM SPSS Statistics (Version 29; IBM Corp.) and R, Version 4.3.0 (R Core Team, 2023).

#### Participants

Our final sample comprised *N* = 152 participants. The average effect size for the effects based on intersecting regularities was around *d*_*Z*_ = 0.43 for the indirect measure and around *d*_*Z*_ = 0.29 (i.e., ~ 0.30) for the explicit measure (Mattavelli et al., 2017, 2023; Richetin et al., 2016). Since we expected the indirect and explicit measures to reverse between both polarity conditions, we planned for a between-participants effect of *d* = 0.60. Therefore, we needed *N* = 148, given α = 0.05, with a power of 1 - β = 0.95 (calculated with G*Power; Faul et al., 2009). We collected data from *N* = 160 participants on Prolific to account for possible dropouts. Only people living in the USA or UK were invited to participate and they had to be fluent in English (we applied this criterion in all further experiments). We excluded four participants because they responded in less than 300 ms on more than 10% of the critical IAT trials (Greenwald et al., 2003). Three participants were excluded because they answered at chance level in the brand categorization task and one participant answered at chance level in the intersecting regularities task.^2^

In the current and the second experiment, we obtained the socio-demographic data from the information provided by the participants on Prolific. Median age was 30 years (and ranged from 19 to 40; one participant did not disclose their age); 98 participants identified as cisgender men, 51 as cisgender women and three participants did not disclose their gender.

#### Design

Experiment 1 comprised a 2-group (plus polarity: *swim* vs. *fly*) between-participants design for the indirect measure. For the explicit measure, the experiment comprised a 2 (plus polarity: *swim* vs. *fly*) x 2 (brand: swim-brand vs. fly-brand) design. The first factor was varied between participants, the second factor within.

#### Materials

##### Animal categorization task

In all tasks using swim/fly words, stimuli were presented in white Arial font at the center of a grey background. Participants responded by pressing the “d” or “k” key. Five swimming animals (“seal,” “dolphin,” “fish,” “whale,” “shark”) and five flying animals (“bird,” “hawk,” “eagle,” “crow,” “parrot”) were used. Each trial began with a fixation cross for 400 ms, followed by the word for 150 ms. Response labels (“can swim”/“cannot swim” or “can fly”/“cannot fly”) were displayed at the bottom of the screen. Trials proceeded only after a response, with a 500 ms inter-trial interval. Erroneous responses triggered feedback (“wrong answer”) for 500 ms. After a four-trial practice phase, participants completed two blocks of 20 trials (each word presented four times). Key assignments were swapped after the first block

##### Brand categorization task

In tasks using Ambio and Ecove products, pictures of five products per brand were displayed at 7 cm × 7 cm in the center of the screen. Each Ambio product was paired with an Ecove product from the same category (e.g., Ambio pasta and Ecove pasta). Participants responded using “d” or “k,” with key assignments counterbalanced. Response labels (“Ambio” and “Ecove”) were displayed at the bottom of the screen. After a four-trial practice phase, each product was presented four times, resulting in two blocks of 20 trials. Keys were swapped after the first block.

##### Intersecting regularities task

Swim/fly words and Ambio/Ecove products were presented together. After a four-trial practice phase, participants completed 160 trials divided into four blocks of 40 trials. One brand and the swim words shared one response key, while the other brand and fly words shared the other key (“d” or “k”). After the first block, key assignments were switched (e.g., Block 1: swim words + Ambio = “d,” fly words + Ecove = “k”; Block 2: swim words + Ambio = “k,” fly words + Ecove = “d”). Trial procedure was identical to previous tasks.

##### IAT

Participants categorized positive and negative words as well as Ambio and Ecove products. Positive words were “happy,” “joy,” “nice,” “paradise,” and “positive”; negative words were “hell,” “negative,” “pain,” “ugly,” and “sad.” Five new products per brand were used, matched pairwise. The IAT procedure consisted of five blocks: Block 1, brand products; Block 2, positive/negative words; Block 3, combined categorization; Block 4, brand products with switched key assignment; Block 5, combined categorization with new key assignment. Practice blocks included 20 trials; crucial blocks (3 and 5) included 80 trials each. The IAT effect was calculated using D (Greenwald et al., 2003). Positive values indicate a preference for the swim brand; negative values indicate a preference for the fly brand.

##### Explicit Measure

At the end of the experiment, participants rated the logo and a set of five products for each brand on a 7-point scale across four dimensions: ugly – nice, worthless – valuable, unpleasant – pleasant, and repulsive – attractive. Each brand was rated eight times (logo + product set × four dimensions), resulting in a total of 16 trials.

#### Procedure

The study was conducted online. Participants could participate using a desktop or laptop computer. The experiment was built with PsychoPy3 and its built-in online translation PsychoJS (Peirce et al., 2019). We used the *ScreenScale* code to ensure a consistent stimulus size across participants (Morys-Carter, 2023). The data were collected via Pavlovia. At the beginning of the experiment, participants were told that they would complete five tasks. In the first task, animal names were presented. Half of the participants were instructed to indicate whether the animals could swim (swim = + condition); the other half indicated whether they could fly (fly = + condition). In the second task, participants saw different products of Ambio and Ecove and their task was to correctly classify both brands to get familiar with them. They then conducted the IR task, where we again used swim/fly words and Ambio/Ecove products. Assignment of the two brands to the different word lists was counterbalanced. After completing the IR task, participants completed an IAT (here we used positive and negative words as well as brand products). In the end we also measured participants’ explicit preferences. After this, participants were debriefed and the experiment was finished.

### Results

#### IAT

We computed D scores for the swim = + and for the fly = + condition based on the improved scoring algorithm by Greenwald et al. (2003). A positive D score indicates preferences for the swim brand, a negative D score a preference for the fly brand. Table 1 shows the mean D values. We found a positive D score in the swim = + condition, *t*(78) = 2.48, *p* = .015, *d*_***z***_ = 0.28, 95% CI [0.05, 0.50]. The D score in the fly = + condition was descriptively negative but not significant, *t*(72) = -1.89, *p* = .063, *d*_***z***_ = − 0.22, 95% CI [-0.45, 0.01]. Most importantly and decisively for our hypothesis, both effects differed significantly from each other, *ΔM* = 0.21, *t*(150) = 3.05, *p* = .003, *d* = 0.50, 95% CI [0.17, 0.82].^3^

**Table 1:** Mean scores for the indirect and explicit measures across conditions for experiment 1 to 4

Measure			Plus Polarity	
Experiment	Ind. Mea.	Dimensions	Swim/self	Fly/other
Indirect Measure				
Experiment 1	IAT	swim – fly	0.12 (0.05)	-0.09 (0.05)
Experiment 2	IAT	self – other	0.10 (0.04)	-0.03 (0.05)
Experiment 3	AMP	swim – fly	0.02 (0.02)	-0.00 (0.01)
Explicit Measure	after			
Experiment 1	[IAT]	swim – fly	0.24 (0.14)	-0.06 (0.12)
Experiment 2	[IAT]	self – other	0.28 (0.14)	-0.03 (0.15)
Experiment 3	[AMP]	swim – fly	0.05 (0.15)	-0.18 (0.14)
Experiment 4	[AMP]	self – other	0.22 (0.15)	-0.11 (0.14)
Overall			0.20 (0.07)	-0.09 (0.07)

Note. For the explicit measure we calculated differences between the mean evaluation of the swim brand and the mean evaluation of the fly brand in Experiment 1 and 3 and between the mean evaluation of the self- and other brand in Experiment 2 and 4. In Experiment 1 and 3, a positive score indicates a preference for the swim brand over the fly brand and a negative score a preference for the fly brand over the swim brand. In Experiment 2 and 4, a positive score indicates a preference for the self brand and a negative score a preference for the other brand. Standard errors of the mean are shown in parentheses

#### Explicit measure

Based on the answers on the 7-point scale, we calculated average values across the four dimensions for the two brands (swim brand vs. fly brand). We conducted a mixed ANOVA with the factors plus polarity (*swim* vs. *fly*) as between-participants factor and brand (*swim-brand* vs. *fly-brand*) as within-participants factor. The two main effects did not reach significance, both *F*’s < 1. Also, the decisive two-way interaction was not significant, *F*(1, 150) = 2.49, *p* = .116, η_p_^2^ = 0.02.

We also calculated rating differences by subtracting explicit ratings for the fly brand from explicit ratings for the swim brand (i.e., positive values indicated a preference for the swim brand over the fly brand) for both polarity conditions. In the swim = + condition the difference was not significant, *M*_*diff*_ = 0.24 (*SE* = 0.14), *t*(78) = 1.70, *p* = .093, *d*_***z***_ = 0.19, 95% CI [-0.03, 0.41]. In the fly = + condition the difference was descriptively negative but also not significant, *M*_*diff*_ = − 0.06 (*SE* = 0.12), *t*(72) = − 0.45, *p* = .652, *d*_***z***_ = − 0.05, 95% CI [-0.28, 0.18].

### Discussion

In Experiment 1 we demonstrated that polarity coding influences IAT measures. When participants coded swim words as + and fly words as –, the resulting D-score was positive, indicating a preference for the swim brand. Conversely, when swim words were coded as – and fly words as +, the IAT effect reversed. The difference between these two IAT effects was statistically significant, supporting our hypothesis. This pattern is compatible with the hypothesis that polarity coding transfers from the stimulus lists to the paired brands via intersecting regularities and subsequently influences IAT performance.

The critical analysis of explicit preference ratings was not significant. Descriptively, there was a preference for the swim brand in the swim = + condition, and this trend reversed in the fly = + condition; however, neither the simple effects within the polarity conditions nor the critical interaction reached statistical significance.

In Experiment 2, we utilized a self–other (SR) task instead of swim versus fly words. Participants discriminated between self-relevant and other-relevant stimuli, with polarity coding manipulated between subjects. Self-relevant stimuli typically serve as a salient reference category and are therefore coded as + by default (Pauly & Wentura, 2025). This default coding is reflected in the self = + condition, where participants categorized stimuli according to self-relevance. In the other = + condition, participants were instructed to categorize stimuli based on other-relevance, thereby defining the other-relevant category as the reference and opposing the normative default. If the manipulation is fully effective, other-relevant stimuli should be + and self-relevant stimuli –, but even partial success allows us to tentatively attribute differences between conditions to polarity changes, because an a priori asymmetry may not be reversed by instructions in all participants.

In the subsequent IAT, the block where the brand paired with the self and positive words shared a response key was defined as the compatible block, whereas the block where the self brand and negative words shared a key was defined as incompatible. Consequently, positive IAT scores indicate an implicit preference for the brand paired with the self, and negative scores indicate a preference for the brand paired with the other.

We expected a positive IAT score in the self = + condition, as both the self-brand and positive stimuli are +, facilitating responses in the compatible block. In the other = + condition, we expected a significant attenuation of the IAT effect. In this latter condition, other-relevant stimuli were task-defined as + and self-relevant stimuli as –, meaning that the structural polarities in the compatible block no longer aligned with typical IAT associations. While a complete reversal of the IAT effect was possible (as observed in Experiment 1), we primarily anticipated a reduction, given that self-relevant stimuli usually constitute a strong reference category and may still be treated as + by some participants. Notably, an alternative interpretation based on positive self-esteem transfer to the brand would predict comparable positive effects in both conditions, which contrasts with the polarity-based expectation of a reduced effect in the other = + condition.

## Experiment 2

### Method

#### Transparency and openness

The general statement about transparency and openness made for Experiment 1 also applies to Experiment 2. The preregistration for Experiment 2 is available under: https://aspredicted.org/gwmj-4hsw.pdf.

#### Participants

Our final sample size comprised *N* = 161 participants. The median age was 33.5 years (range: 18–50), based on the 160 participants who provided age information; one participant did not disclose their age. Ninety-one participants identified as cisgender men and 69 as cisgender women and one participant did not disclose their gender. We again estimated our sample size based on effect sizes of around *d*_*Z*_ = 0.43 for the IAT and around *d*_*Z*_ = 0.29 for the explicit measure (Mattavelli et al., 2017, 2023; Richetin et al., 2016). We expected to replicate the IR effect in the self = + condition. Therefore, we needed *N* = 75, given α = 0.05 (one-tailed), to achieve a power of 1 - β = 0.80 (calculated with G*Power; Faul et al., 2009). However, to satisfy the counterbalancing requirements we needed *N* = 96 (which yielded a planned power of 1- β = 0.88 for *d*_*Z*_ = 0.29). Since we needed the same number of participants in both polarity conditions, we recruited a total sample of *N* = 192.

Crucially, for our primary hypothesis-testing analysis—the comparison of the IR effect between the self = + and other = + conditions—the total sample size of *N* = 192 (= 2 × 96) provided a robust power range: It provided a power of 1 - β = 0.80 to detect a scenario where the IR effect in the other = + condition was merely reduced (by d = 0.36). It provided a power of up to 1 - β = 0.99 if the effect completely reversed (as in Experiment 1, which we also considered possible). Thus, our power planning in Experiment 2 was designed to cover a plausible range of effect sizes, ensuring that statistical power was at least 0.80 for a conservative reduction scenario of obtaining only an effect reduction in the other = + condition, and well above 0.95 for the anticipated reversal effect.

We excluded the data of 31 participants. Two participants answered faster than 300 ms in more than 10% of the IAT trials, 26 participants answered at chance level in matching trials or the brand categorization task, and three participants answered the control question (see below) incorrectly. With *N* = 161 participants (*n* = 78 and *n* = 83 for the polarity conditions) we had a power of 1- β = 0.84 (α = 0.05, one-tailed) to replicate the IR effect in the self = + condition with α = 0.05 (one-tailed), a power of 1- β = 0.98 (α = 0.05, one-tailed) to find a full reversal of *d* = 0.58 (= 0.29 – [-0.29]), and a power of 1- β = 0.80 (α = 0.05, one-tailed) to find a partial reversal of *d* = 0.39.

#### Design

Experiment 2 comprised a 2-group (plus polarity: *self* vs. *other*) between-participants design for the indirect measure. For the explicit measure, the experiment comprised a 2 (plus polarity: *self* vs. *other*) x 2 (brand: self-brand vs. other-brand) design. The first factor was varied between participants, the second factor within.

#### Materials

##### Matching task

After participants associated themselves and another person with a geometric shape (shape assignment was counterbalanced between participants), they were told that in the first task one label (either “You” or “She”) and one shape were presented on the screen and they had to react to whether the displayed label-shape combination matched (both label and shape referred to the same person) or mismatched (shape and label did not refer to the same person). Shapes were either a square or a circle. Each shape could be presented in five different colors (colors of the shapes were irrelevant for the task). Participants were instructed to respond by pressing “d” in matching trials and “k” in non-matching trials. Each trial began with presentation of a fixation cross in the middle of the screen for 200 ms. After a blank of 100 ms the label-shape combination was presented for 150 ms. From stimulus onset participants had 2000 ms to respond. The inter-trial interval was 300 ms. If participants responded too slowly or incorrectly, they received error feedback. After a practice phase of eight trials, participants completed two blocks of 20 trials each. After each block, participants answered a control question asking them to indicate which shape referred to themselves and which shape referred to the other person. This was the control question used for the exclusion criterion reported in the Participants section.

##### Brand categorization task

This task was similar to the task in Experiment 1, except for one minor detail: A red cross (instead of the text “wrong answer”) appeared after participants gave an erroneous response and participants were told to give the right response before continuing with the next trial. After a practice phase of four trials, two blocks of 10 trials each followed.

##### Intersecting-regularities task

In this task, one half of the participants was instructed to categorize whether geometric shapes were self-relevant or not, and the other half was instructed to categorize whether shapes were other-relevant or not. Moreover, participants were told to react to Ambio and Ecove products using the same keys as for the shapes. For example: “If you see a product with the logo of Ambio, press the ‘d’ key and if you see a product with the logo of Ecove, press the ‘k’ key.” Further: “If you see a shape that represents yourself (= circle), press the ‘k’ key, otherwise press the ’d’ key.” Assignment of brands and shapes to the keys was counterbalanced. The trial procedure and number of trials were the same as in Experiment 1, except that stimuli remained on the screen until a response was made. A red cross appeared following an incorrect response and remained on the screen until the correct response was made.

##### IAT and explicit measure

The IAT procedure was identical to Experiment 1. We again measured IAT effects for both polarity conditions (self = + vs. other = +) by calculating D. This time a positive IAT score indicates preferences for the self brand, whereas a negative score indicates preferences for the other brand. The explicit measure was the same as in Experiment 1.

#### Procedure

The experiment was identical to Experiment 1, with the following differences. At the beginning of the experiment participants learned to associate themselves with a geometric shape (either circle or square) and another person with another geometric shape. This time their first task was to categorize stimuli with regard to their self-relevance. Therefore, participants completed a matching task based on Sui et al. (2012). We used this task for participants to learn to associate one geometric shape with themselves and one geometric shape with another person. Afterwards they categorized Ambio/Ecove products as in Experiment 1. In the SR task participants saw either products of both brands or the geometric shapes. Here we varied instructions between participants: One half of the participants were instructed to react to whether the geometric shapes referred to themselves or not. The other half were instructed to react to whether the geometric shapes referred to others or not. IAT and the explicit measure were the same as in Experiment 1.

### Results

#### IAT

We computed D scores for the self = + and for the other = + condition based on the improved scoring algorithm by Greenwald et al. (2003). A positive D score indicates preferences for the self brand, a negative D score a preference for the other brand (see Table 1). We found a positive D score in the self = + condition, *t*(82) = 2.62, *p* = .010, *d*_***z***_ = 0.29, 95% CI [0.07, 0.51]. The D score in the other = + condition was descriptively negative but not significant, *t*(77) = − 0.63, *p* = .533, *d*_***z***_ = -0.07, 95% CI [-0.29, 0.15]. Most importantly both effects differed significantly from each other, *ΔM* = 0.14, *t*(159) = 2.25, *p* = .026, *d* = 0.35, 95% CI [0.04, 0.67].^4^

#### Explicit measure

Based on the answers on the 7-point scale, we calculated average values across the four dimensions for the two brands (self brand vs. other brand). We conducted a mixed ANOVA with the factors plus polarity (*self* vs. *other*) as between-participants factor and brand (*self-brand* vs. *other-brand*) as within-participants factor. The main effect of brand did not reach significance, *F*(1, 159) = 1.50, *p* = .222, η_p_^2^ = 0.01. Neither the main effect of plus polarity, *F* < 1, nor the critical two-way interaction, *F*(1, 159) = 2.23, *p* = .138, η_p_^2^ = 0.01, was significant.

We calculated differences between the self- and other brand by subtracting ratings for the other brand from the self brand. Therefore, positive scores indicated a preference for the self brand. The self brand was rated significantly more positively in the self = + condition, *t*(82) = 2.04, *p* = .045, *d*_***z***_ = 0.22, 95% CI [0.01, 0.44]. In the other = + condition there was no significant difference, *t*(77) = -0.18, *p* = .859, *d*_***z***_ = -0.02, 95% CI [-0.24, 0.20].

### Discussion

In Experiment 2 we successfully replicated the core pattern from Experiment 1. The IAT scores were significantly higher in the self = “+” condition than in the other = “+” condition. Because the only difference between the two experimental groups was the task-defined reference category, this difference directly supports a polarity account. Under this account, in the self = + condition, participants would encode self brand as + and other brand stimuli as –, which facilitates responses in the IAT block where both + coded categories (self brand, positive words) share a response key. In the other = + condition, the self-relevant category was task-defined as –, meaning that the categories sharing a key in the compatible block (self brand [–] / positive words [+]) no longer possessed corresponding polarities, which slowed down responses. Instead, polarity correspondence emerged in the block defined as incompatible (self brand [–] / negative words [–]; other brand [+] / positive words [+]), systematically driving the observed attenuation of the IAT effect.

For the explicit measure, there was again no significant difference between the self = + and other = + conditions. However, the pattern of means aligned with the hypothesis: in the self = + condition, liking for the self brand was significantly higher than for the other brand, whereas in the other = + condition this effect was descriptively reversed, but the difference was not significant. Note that, with our self = + condition we replicated the results by Richetin et al. (2016; see also Mattavelli et al., 2019, 2021; Perkins & Forehand, 2012; Prestwich et al., 2010) for the IAT as well as for the explicit rating.

## Experiment 3

Experiment 3 was conceptually analogous to Experiment 1, but we used the Affect Misattribution Procedure (AMP; Payne et al., 2005) instead of the IAT. Unlike the IAT, which can be directly interpreted in terms of polarity correspondences without invoking underlying valence changes, alternative indirect measures and explicit ratings require an additional theoretical assumption: manipulating category polarities may induce subtle valence differences. For instance, if Ambio is coded as + and Ecove as –, Ambio may be slightly preferred over Ecove as a secondary consequence of this asymmetric framing. The critical tests of the manipulation on explicit ratings in Experiments 1 and 2, however, were not significant.

In contrast, the AMP is not expected to be influenced by polarity correspondences. If polarity manipulations directly alter stimulus valence, however, we should observe a positive AMP effect in the swim = + condition and a negative effect in the fly = + condition (AMP scores were computed by subtracting the proportion of pleasant responses following fly-brand primes from the corresponding proportion following swim-brand primes). Corresponding differences might also emerge in explicit ratings.

### Method

#### Transparency and openness

The general statement about transparency and openness made for Experiment 1 also applies to Experiment 3. The preregistration for Experiment 3 is available under: https://aspredicted.org/kwf7-vqnp.pdf.

#### Participants

Our final sample size comprised *N* = 145 participants. We had the same power considerations as in Experiment 1. In total we collected the data of *N* = 189^5^ We excluded the data of 44 participants. 21 participants were excluded because they answered too fast (< 150 ms) or too slow (> 3000 ms) in more than 40% of the AMP trials. 10 participants were excluded because they used the same key in more than 90% of the trials in the AMP. Twelve participants were excluded because they answered at chance level in at least one of the reaction time tasks. And one participant was excluded because mean presentation times for stimuli during the AMP were not accurate.^6^Median age of the final sample was 32 years (and ranged from 19 to 40), 82 participants identified as cisgender men and 62 as cisgender women and one participant did not disclose their gender.

#### Design

Experiment 3 comprised a 2-group (plus polarity: *swim* vs. *fly*) between-participants design for the indirect measure. For the explicit measure, the experiment comprised a 2 (plus polarity: *swim* vs. *fly*) x 2 (brand: swim-brand vs. fly-brand) design. The first factor was varied between participants, the second factor within.

#### Procedure and materials

The procedure and materials were the same as in Experiment 1, except that we used an AMP as the indirect measure.

##### AMP

The setup was based on the procedure introduced by Payne et al. (2005). Participants were instructed to rate the pleasantness of the Chinese characters while ignoring the primes. They were explicitly informed that the prime was only a warning signal for the following Chinese character. A standard trial was as follows: The prime was presented for 80 ms in the center of the screen, followed by a blank screen for 120 ms and then a Chinese character for 100 ms. Finally, a mask (black and white “noise”) replaced the Chinese character and remained on the screen until the participant categorized the character with regard to pleasantness (i.e., more or less pleasant than the average character). Participants responded using the “c” key for pleasant responses and the “m” key for unpleasant responses. The next trial started as soon as participants responded. In the beginning, there was a practice phase of five trials. This was followed by the experimental phase, which consisted of 75 trials. In one third of the trials, the prime was an image representing the brand *Ambio*, in one third an image representing the brand *Ecove* and in one third a neutral image (a grey square). Five different images were used for both *Ambio* and *Ecove*. One of the five images was always the brand logo and the other four were images of products. Note that the product images used were not the same as those used in the familiarization and SR tasks (see Sect.  2). The product categories used in this task were: pasta, milk, tea, and cookies.

### Results

#### AMP

We computed the proportion of pleasant ratings for each brand and subtracted the mean pleasant ratings for the fly brand from those for the swim brand. A positive score indicates general preferences for the swim brand, a negative score a preference for the fly brand (see Table 1). We found a positive but not significant score in the swim = + condition, *t*(73) = 0.86, *p* = .391, *d*_***z***_ = 0.10, 95% CI [-0.13, 0.33]. The score in the fly = + condition was descriptively negative but also not significant, *t*(70) = − 0.26, *p* = .798, *d*_***z***_ = -0.03, 95% CI [-0.26, 0.20]. Both effects did not differ significantly from each other, *ΔM* = 0.02, *t*(143) = 0.85, *p* = .399, *d* = 0.14, 95% CI [-0.19, 0.47].

#### Explicit measure

We conducted a mixed ANOVA with the factors plus polarity (*swim* vs. *fly*) as between-participants factor and brand (*swim-brand* vs. *fly-brand*) as within-participants factor. The main effects did not reach significance, both *F’s < 1.* The decisive two-way interaction was also not significant, *F*(1, 143) = 1.32, *p* = .252, η_p_^2^ = 0.01. Further, there was no significant difference between both brands in the swim = + condition, *t*(73) = 0.36, *p* = .718, *d*_***z***_ = 0.04, 95% CI [-0.19, 0.27], and also none in the fly = + condition, *t*(70) = -1.28, *p* = .205, *d*_***z***_ = -0.15, 95% CI [-0.39, 0.08].^7^

### Discussion

In Experiment 3, neither the indirect measure nor the explicit ratings showed a significant difference between the swim = + and fly = + conditions. Neither the within-condition AMP tests nor the between-condition comparison was significant. There were also no effects for the explicit measure although the descriptive differences were in the hypothesized direction: participants tended to evaluate the swim brand as more positive in the swim = + condition and the fly brand as more positive in the fly = + condition. Although up to now none of the experiments showed the hypothesized effect in explicit ratings, the descriptive pattern was consistent across experiments. We postpone further discussion until after Experiment 4.

## Experiment 4

Experiment 4 was a conceptual replication of Experiment 2 without the IAT, as in Experiment 3. We again tested whether hypothesis-consistent explicit ratings would emerge without a preceding IAT. As in Experiment 3, the explicit ratings were obtained after an AMP-like task, which should, however, be solely regarded as a filler task in Experiment 4.^8^

### Method

#### Transparency and openness

The general statement about transparency and openness made for Experiment 1 also applies to Experiment 4. The preregistration for Experiment 4 is available under: https://aspredicted.org/cz2g-ymff.pdf.

#### Participants

Our final sample comprised *N* = 118 participants. We collected the data from *N* = 163 participants. We had the same power considerations as in Experiment 2. Unfortunately, we had to discard more participants than initially thought. We excluded the data of 45 participants. 29 participants were excluded because they answered at chance level during the matching task. One additional participant was excluded because performance was at chance level in the brand categorization task. Six participants answered too fast (< 150 ms) or too slow (> 3000 ms) in more than 40% of the AMP trials. Nine participants were excluded because they used the same key in more than 90% of the trials in the AMP.^9^However, with our sample size we still had a power of 1 - β > 0.90, to detect a reversal of the SR effect from the self = + to the other = + condition (i.e., an effect of *d* = 0.58), given α = 0.05 (one-tailed). Median age of the final sample was 34.5 years (and ranged from 18 to 50), 76 participants identified as cisgender men and 38 as cisgender women and 4 participants did not disclose their gender.

#### Design

Experiment 4 comprised a 2 (plus polarity: *self* vs. *other*) x 2 (brand: self-brand vs. other-brand) design for the explicit measure. The first factor was varied between and the second within participants.

#### Procedure and materials

Procedure and materials were equivalent to Experiment 2 with the only exception that we did not use the IAT as an indirect measure. Instead, participants worked through an AMP-like task (see Experiment 3) before responding to the explicit ratings. As noted above, the AMP-like task was treated as a filler task in this experiment.

### Results

#### Explicit measures

Again, we conducted a mixed ANOVA with the factors plus polarity (*self* vs. *other*) as between-participants factor and brand (*self-brand* vs. *other-brand*) as within-participants factor. The main effects of brand and plus-polarity did not reach significance, both *F’s < 1.* The decisive two-way interaction also did not reach significance, *F*(1, 116) = 2.68, *p* = .104, η_p_^2^ = 0.02. Again, positive scores indicated a preference for the self brand. The difference between ratings of the self and other brands was not significant in the self = + condition, *t*(52) = 1.49, *p* = .143, *d*_***z***_ = 0.21, 95% CI [-0.07, 0.48]. In the other = + condition, the difference was also not significant, *t*(64) = -0.80, *p* = .424, *d*_***z***_ = -0.10, 95% CI [-0.34, 0.14].

### Discussion

Again, we found no significant effect in the explicit ratings, although the data descriptively aligned with our predictions: positive rating differences in the self = + condition and negative ratings in the other = + condition. Remarkably, all four experiments exhibited a trend consistent with our hypothesis (see Table 1). This suggests that polarity may influence explicit ratings, albeit modestly.

When multiple experiments show consistent trends but weak statistical evidence, it is recommended to leverage the full available data. Goh and colleagues (2016) proposed a “mini meta-analysis” for such cases; if raw data are accessible, a “mega-analysis” is even more informative (e.g., Eisenhauer, 2021; Scoboria et al., 2017), as it pools the data across experiments to increase statistical power. Accordingly, we conducted a cross-experimental analysis to test for an overall effect of polarity on explicit ratings using the full dataset.^10^

## Cross-experimental analysis of explicit ratings

To test for an overall effect of polarity on explicit ratings, we conducted an ANOVA with the factors plus polarity (*swim/self* = + vs. fly/other = +), type of preceding indirect measure (IAT vs. AMP), and source category (swim/fly vs. self/other) as between factors and the rating differences between the swim and fly brands in Experiments 1 and 3, or between the self and other brands in Experiments 2 and 4 (see Table 1) as the dependent variable. In this ANOVA the main effect of plus polarity was significant, *F*(1, 568) = 8.33, *p* = .004, η_p_^2^ = 0.014 (*d* = 0.24). This effect was neither moderated by the type of indirect measure, by the source category, nor by the type of indirect measure × the source category interaction, all *Fs* < 1. Thus, across experiments there was a small effect of polarity-induced valence connotation, which shows neither dependency on the source (i.e., whether swim/fly or self/other were used in the polarity induction) nor on the type of preceding indirect measure (it does not depend on a preceding IAT).

To obtain an overall impression of the evidence for an effect of polarity on our explicit measures, we additionally conducted supplementary Bayesian analyses that were not preregistered. We calculated difference scores between explicit ratings for the plus-polar and minus-polar brand, with positive values indicating a preference for the brand associated with the + polar source category. Using the BayesFactor package in R, we conducted one-sample JZS Bayesian t-tests on these scores (*N* = 576; Rouder et al., 2009). Both analyses used a Cauchy prior on the standardized effect with scale r = √2/2 (approximately 0.707). The unrestricted alternative, allowing positive or negative effects, yielded BF10 = 3.13. The directional alternative, restricted to positive effects, yielded BF + 0 = 6.24. According to the classification scheme proposed by Wagenmakers et al. (2011), both results favor their respective alternative hypotheses with “substantial evidence” (i.e., relative to the point null hypothesis in the first case and to the complementary negative-or-zero-effect hypothesis in the second).

The analyses did not include the additional factors in the pooled ANOVA and should not be regarded as its Bayesian equivalent, because the models and the parameters to which their priors apply differ. Considered alongside the pooled frequentist result and the non-significant critical tests in the individual experiments, the supplementary analyses provide cautious evidence for a small explicit evaluation effect.

The ANOVA provided further information. The intercept test of the analysis indicates whether there is a significant asymmetry between the two polarity conditions in the strength of the effect (i.e., whether the effect of M = 0.20 is larger than the effect of M = -0.09, direction ignored; see last row of Table 1 for the values).^11^This test was not significant, *F*(1, 568) = 1.07, *p* = .302, η_p_^2^ = 0.002. Neither the main effect of preceding measure, the main effect of source category, nor their interaction was significant, all *Fs* < 1.26. That is, with regard to inferential statistics there are no asymmetries.

However, one might argue that the question of asymmetry means something fundamentally different for the swim/fly and self/other experiments. For the swim/fly experiments, an asymmetry would simply mean that the materials are a bit unbalanced (e.g., swim items might be on average more positive than fly items or vice versa). For the self/other experiments, an asymmetry would mean either that the a priori polarity of self +/other – cannot be fully changed by instruction or that the positivity of the self induces valence changes beyond polarity. Especially the latter possibility is of interest.

Therefore, despite the non-significance of the main effect of source category in the analysis reported above, we repeated the analysis separately for the swim/fly experiments and the self/other experiments, respectively. For the swim/fly experiments, the intercept test was not significant, *F* < 1, and there was no main effect of type of preceding indirect measure, *F*(1,293) = 1.30, *p* = .256, η_p_^2^ = 0.00. That is, there were no indications of imbalances of the swim/fly materials.

For the self/other experiments, the intercept test was not significant as well, *F*(1,275) = 1.50, *p* = .222, η_p_^2^ = 0.005; *F* < 1 for the main effect of type of preceding indirect measure. That is, the intercept test provided no indication that the self category induced positive valence beyond polarity. However, for the sake of transparency, the mean difference in the self = + condition was significantly different from zero, *F*(1,134) = 5.76, *p* = .018, η_p_^2^ = 0.041; *F* < 1 for the main effect of type of preceding indirect measure, whereas the corresponding mean difference in the other = + condition was not significantly different from zero, *F* < 1; *F* < 1 for the main effect of type of preceding indirect measure.

## General discussion

We investigated whether polarity coding of source categories can influence indirect and explicit measures after an intersecting regularities (IR) task. Given that the IAT is commonly used after IR tasks and is susceptible to polarity coding (Proctor & Xiong, 2015; Rothermund & Wentura, 2004), we hypothesized that observed task-elicited effects may reflect structural polarity alignment rather than genuine valence transfers. In Experiment 1, we utilized two normatively neutral word lists as source categories: animals that can swim and animals that can fly. Within the IR task, each list was paired with a specific brand. Subsequent implicit evaluations were assessed via an IAT, where positive scores indicated a preference for the brand paired with the swim list (swim brand) and negative scores indicated a preference for the brand paired with the fly list (fly brand). We manipulated polarity coding of the source categories between participants. Half of the participants processed swimming animals as the reference category and coded these stimuli as + (with flying animals as –), while the other half used flying animals as the reference category encoding them as + (with swimming animals as –). Results showed a positive IAT score in the swim = + condition and a negative score in the fly = + condition; this difference was statistically significant.

In Experiment 2, we applied a conceptually parallel manipulation using self- and other-relevant stimuli as source categories. Typically, the positivity associated with the self leads to a preference for the brand linked to the self (self brand) over the brand linked to another person (other brand) in the IAT. Here, we aimed to test whether the IAT effects could instead be explained by which category is processed as the reference category.

We manipulated whether the self-category served as the reference category (self [+], other [–]) or the other-category served as the reference (other [+], self [–]). IAT effects were computed such that positive scores indicated a preference for the self brand, and negative scores indicated a preference for the other brand. Results showed a significant positive IAT score in the self = + condition and a negative score in the other = + condition, with the difference reaching significance. Crucially, if IAT performance depended solely on transferring the self’s pre-existing positivity independently of task framing, comparable positive scores would be expected in both conditions.

In Experiment 3, we implemented the Affect Misattribution Procedure (AMP) as an indirect measure instead of the IAT. This change allowed us to test (a) whether effects on explicit ratings depend on the prior administration of an IAT, and (b) whether polarity manipulations directly alter the valence of target categories rather than only influencing IAT measures. In this experiment, we found no significant differences between the swim = + and fly = + conditions on the AMP.

Across all experiments, we also measured explicit evaluations for both brands. While the individual experiments did not yield statistically reliable evidence that explicit ratings were modulated by polarity coding, the descriptive data consistently indicated that brands associated with the “+” polarity source category were evaluated more favorably than brands associated with the “-” polarity source category. The pooled analysis yielded a small effect (*d* = 0.24, *p* = .004). Supplementary Bayesian analyses yielded *BF*10 = 3.13 for the unrestricted alternative and *BF* + 0 = 6.24 for the positive directional alternative. Because the cross-experimental and Bayesian analyses were not preregistered, we regard these findings as cautious evidence that polarity framing may influence explicit evaluations. Further research is needed to establish the robustness and mechanism of this effect.

### Implications for direct and indirect measures in IR tasks

The findings show that task-defined polarity framing influenced subsequent IAT performance in Experiments 1 and 2. This effect is compatible with the hypothesis that asymmetric processing of source categories transfers to the paired brands and affects IAT performance through polarity correspondence. This seems particularly relevant for studies using self-other discriminations and indirect measures such as the IAT. We assume that, in self-referencing (SR) tasks, the self-category is typically treated as the + category (see also Pauly & Wentura, 2025). Consequently, a brand paired with the self-category via intersecting regularities is also coded as +. In the standard IAT, a compatible block is defined as one in which self-relevant stimuli and positive words share a response key. If polarity correspondence is present in this block—that is, if both the self-relevant category and positive words are + coded—then the resulting IAT effect could be explained in terms of polarity coding rather than valence. However, the IAT findings do not establish whether this influence was entirely structural (i.e., was solely based on polarity differences) or whether polarity framing also produced evaluative changes that contributed to performance. In particular, the use of positive and negative attribute categories permits both direct structural effects and effects mediated by target evaluations.

For explicit ratings, the critical tests in the individual experiments were not significant, whereas the non-preregistered pooled analysis provided cautious evidence for a small effect. These findings indicate that polarity manipulations could subtly affect explicit measures of preference as well. The analysis across all experiments further indicates that the type of preceding indirect measure (IAT vs. AMP) does not moderate the intersecting regularities effect on explicit ratings, suggesting that the observed differences are not mediated by the IAT. These findings imply that polarity manipulations can directly influence valence. One might note that this conclusion appears inconsistent with the absence of an AMP effect in Experiment 3, which would be expected if polarity manipulations directly changed valence. However, the cross-experimental effect for explicit ratings was very small, and assuming the same effect size for the AMP measure yields a post hoc power of 1–β = 0.30 (two-tailed) to detect the effect in the sample of Experiment 3.

Further, it is well-established that implicit and explicit measures can dissociate, a phenomenon explicitly accounted for by the Associative-Propositional Evaluation (APE) model (e.g., Gawronski & Bodenhausen, 2014). According to this framework, implicit measures capture the spontaneous activation of associative connections, whereas explicit measures rely primarily on propositional reasoning that evaluates the validity and consistency of available information. Consequently, the absence of an effect in the Affect Misattribution Procedure (AMP) does not preclude the emergence of an effect on explicit preference measures. One possible explanation is that polarity framing influenced propositional evaluation without a corresponding change in the associative activation relevant to the AMP.

All in all, it appears that valence differences could be generated through polarity manipulations. However, the differences are rather subtle and need further investigation. Also, the precise mechanism contributing to this effect remains unclear. In the introduction, we provided different approaches to explain a possible effect of polarity manipulations on explicit ratings: We suggested effects of processing fluency (e.g., Reber et al., 1998; Winkielman & Cacioppo, 2001), the possibility that the reference category serves as a salient cognitive anchor that stands out against the contrast category (Mrkva & Van Boven, 2020) or effects of affirmative responding with regard to the reference category. Those accounts, however, represent only an initial step; a comprehensive theoretical framework that fully explains the pathways through which polarity coding modulates valence has yet to be developed.

### Open questions

Two pathways may contribute to the effects of polarity framing on IAT performance. In a direct pathway, the IR task establishes asymmetries between the target categories that facilitate responding when corresponding polarities share a response key (Mechanism A). In an evaluatively mediated pathway, polarity framing changes target evaluations, and these changes contribute to subsequent IAT performance (Mechanism B). The pathways are not mutually exclusive: the same manipulation may produce structural and evaluative changes, and both may influence the IAT. Furthermore, as discussed above, explicit evaluative changes may arise through propositional processing without necessarily producing a detectable AMP effect.

The present findings do not establish which pathway predominated. Significant IAT effects alongside less conclusive explicit and AMP findings do not by themselves show that the IAT effects were mainly structural. The pooled explicit findings leave open an evaluative contribution, while the non-significant AMP result neither confirms nor excludes it.

Future research could use non-evaluative attribute categories, such as words versus non-words, in place of positive and negative words in the IAT (Rothermund & Wentura, 2004). If the effect of polarity framing persisted, this would strengthen a structural interpretation by reducing the role of explicitly evaluative attribute categories. Such a design should be combined with independent assessments of target evaluation and category asymmetry, rather than treating persistence of an IAT effect as unequivocal evidence of a single process.

Experiment 2 nevertheless shows that changing the task-defined reference category influenced later IAT performance while the identity of the self- and other-related source categories remained unchanged. This finding challenges an account in which transfer of a fixed, pre-existing positivity of the self alone determines the effect independently of task framing. It does not, however, distinguish a direct influence of polarity correspondence from evaluative changes induced by that framing.

### Implications for other studies

Our results also provide a further explanation for a study by Mattavelli et al. (2023). They conducted several experiments using a self-referencing (SR) task and compared different source categories. In a series of experiments employing a self-referencing (SR) task, these authors systematically varied the valence and self-relevance of their source categories. Specifically, Experiment 1 contrasted positive self-relevant words with positive other-relevant words. Experiments 2 and 3 compared negative self-relevant words with negative other-relevant words. Most critically, Experiment 4 contrasted negative self-relevant words directly with positive other-relevant words. Across all subsequent IATs, participants consistently demonstrated an implicit preference for the brand paired with the self via intersecting regularities.

Experiment 4 is particularly noteworthy, as the preference for the self brand cannot be explained by valence transfer in the SR task—here, self-adjectives were negative, while other-relevant adjectives were positive. Based on the empirical evidence presented in the current paper, we suggest that these seemingly paradoxical results reflect the cognitive dominance of the self-category as the normative reference point. That is, across all four experiments, participants likely processed the stimuli based on a structural feature asymmetry—namely, whether the words were self-referential (+) or not (-)—independent of the nominal valence of the source categories. Thus, structural asymmetries associated with self-relevance may contribute to the subsequent self-brand advantage, either directly through polarity correspondence or indirectly through evaluative changes induced by asymmetric processing.

### Constraints on generality

We assume that the Polarity Correspondence Principle (Proctor & Cho, 2006) reflects a fundamental principle of human information processing and that the coding of stimuli as having + or – polarity can be regarded as a general cognitive process. However, with regard to Experiments 2 and 4 a potential limitation may be differences in polarity coding between individualistic and collectivistic countries. The participants in our study were drawn from either the US or UK, that is, English-speaking, Westernized, individualistic countries. It is assumed that there are differences between individualistic and collectivistic cultures, particularly with regard to the processing of self-relevant information (e.g., Liu et al., 2015). In collectivistic countries the “other” may play a more important role and therefore may also be used more frequently as a reference category. Thus, in non-individualistic cultures it is possible that the coding of self-referential stimuli as + polarity may be less robust than in individualistic countries (but see Golubickis et al., 2019). Whether the effect generalizes to populations with different cultural orientations remains an open empirical question.

### Conclusion

Across four experiments, we examined the influence of polarity framing in intersecting regularities tasks. Manipulating the task-defined reference category influenced IAT performance, and a non-preregistered pooled analysis provided cautious evidence for a small effect on explicit evaluations. The AMP in Experiment 3 did not yield a significant effect. The IAT findings are compatible with direct polarity correspondence, evaluative changes induced by polarity framing, or both. Explicit judgments may additionally reflect propositional processing of the IR experience. Future research should distinguish these pathways using measures and manipulations that separately assess structural asymmetry and evaluation. The present findings highlight the importance of accounting for polarity framing when interpreting IR effects, while leaving the relative contributions of structural and evaluative processes open.

## Data Availability

Raw data, analysis scripts and experiment files for each experiment can be found at https://osf.io/ev89m.

## Footnotes

1 Readers who have no knowledge about the IAT are referred to the next Section.

2 To be more specific: The cumulative probability of making 14 [15] errors or fewer, when working at chance level, given 40 trials in the brand categorization task (see below), 𝑋 ∼ Binomial (𝑛 = 40, 𝑝 = 0.5), was less [greater] than 5%, P(errors ≤ 14) = .040 [P(errors ≤ 15) = .077]. Therefore, we excluded participants with more than 14 errors in this experiment. We calculated chance level for the other reaction time tasks in this and all further experiments in the same manner (taking into account the number of trials in the respective tasks).

3 We assume that some readers would have expected an alternative analysis: The IAT score could alternatively be coded with reference to the plus polar category such that the mean score in the swim = + condition would still be *M* = 0.12, but the mean score in the fly = + condition would switch sign (i.e., *M* = 0.09). The decisive test would then be whether the overall mean (i.e., *M* = 0.105) is significantly above zero. Note that this test is equivalent to the test presented here (if the factor positive polarity [i.e., swim = + vs. fly = +] is included in the analysis to account for possible differences between the two balancing conditions).

4 Here we deviate (in an uncritical manner) from our preregistered analysis plan for the indirect measure (IAT). We preregistered a mixed-design ANOVA with plus-polarity assignment (self = + vs. other = +) as a between-participants factor and block (“compatible” vs. “incompatible”) as a within-participants factor. However, an independent-samples t-test comparing the IAT effect in the self = + condition with the IAT effect in the other = + condition is mathematically equivalent to the interaction term in the mixed ANOVA (with t = √F). To facilitate the interpretability of our findings and ensure comparability across all experiments, we decided to report our results consistently using the same analytical approach as in Experiment 1.

5 Our original power analysis indicated that a target sample size of N = 148 (noted as 152 due to our counterbalancing requirements) was required to achieve a statistical power of 1 - β = .95 for our expected effect size. To account for exclusions based on our preregistered criteria, we iteratively checked the number of remaining eligible participants after a first data collection of N = 152 participants. We then recruited additional participants as long as necessary. (Thus, we finally collected the data of N = 189 participants.) Data collection was ultimately stopped at N = 145 valid participants because a post-hoc sensitivity check confirmed that this sample size still maintained a power of approximately 1 – β = .95.

6 We determined the frame rate of each participant and calculated the mean presentation duration for the prime stimulus, the blank and the target. Participants for whom the deviation of the participants identified as cisgender men and 62 as cisgender women and one participant did not disclose their gender.

7 Here we deviate again (in an uncritical manner) from our preregistered analysis plan for the explicit measure. We preregistered the use of an independent t-test instead of a mixed ANOVA. The interaction in a mixed ANOVA is mathematically equivalent to a t-test comparing brand preference differences between the two polarity conditions (with t = √F).

8 In fact, Experiment 4 was chronologically the first of all experiments reported in this article. It was planned with an AMP as the indirect measure. However, post hoc it turned out that the AMP data could not be taken as valid because of some programming details (i.e., timing routines that were inadequate for the online assessment). For participants, however, the task was experienced as a typical AMP. Since the explicit ratings can be taken as valid, we decided to report this experiment to prevent a “file drawer” problem.

9 As noted above, the AMP is not part of the data analysis because of post hoc technical problems. Nevertheless, we kept the pre-registered exclusion criteria because we consider them to be general compliance criteria for participants.

10 Vosgerau et al. (2019) discuss how study selection and analytical flexibility can compromise the interpretation of internal meta-analyses. The four experiments reported here comprise all experiments conducted in our laboratory within this research program; no additional experiments were withheld. However, the decision to pool the explicit ratings across experiments was made after the individual results were known, and the pooled analysis and supplementary Bayesian analyses were not preregistered. Including all experiments addresses the concern about omitted studies but does not make these analyses preregistered confirmatory tests. We therefore interpret the pooled findings cautiously and distinguish them from the preregistered tests within the individual experiments.

11 The intercept test tests whether the average difference of M = 0.06 (= [0.20 + (-0.09)]/2) is significantly different from zero. Alternatively, one might reverse the sign of the difference score for the fly/other = + samples and test whether the mean difference (i.e., M = 0.09) is significantly lower than the mean difference for the swim/self = + samples (i.e., M = 0.20), a test that might appear more intuitive. Note, the two tests are equivalent as long as the between-participants factor plus polarity is included in the intercept analysis.

## Code Availability

Analysis scripts are available in the OSF repository linked above.
